# The Cytokine and Chemokine Profiles in Patients with Hand, Foot and Mouth Disease of Different Severities in Shanghai, China, 2010

**DOI:** 10.1371/journal.pntd.0002599

**Published:** 2013-12-19

**Authors:** Mei Zeng, Xiaoyan Zheng, Ruicheng Wei, Na Zhang, Kai Zhu, Bin Xu, Chun-Hui Yang, Chun-Fu Yang, Chaoyang Deng, Dongbo Pu, Xiaohong Wang, Ralf Altmeyer, Qibin Leng

**Affiliations:** 1 Department of Infectious Diseases, Children’s Hospital of Fudan University, Shanghai, China; 2 Key Laboratory of Molecular Virology and Immunology, Institut Pasteur of Shanghai, Shanghai Institutes for Biological Sciences, Chinese Academy of Sciences, Shanghai, China; The George Washington University Medical Center, United States of America

## Abstract

**Background and purpose:**

Systemic upregulation of inflammatory cytokines is characteristic of critical severe hand, foot, and mouth disease (HFMD) with pulmonary edema. Thus, immunomodulatory medicines such as steroids, including methylprednisolone, have been proposed to treat patients with severe HFMD in China, because it is postulated that inflammatory cytokines play a role in the development of severe complications. This study is to further investigate the inflammatory response in the relatively mild HFMD patients, and whether steroid treatment has a beneficial effect on the suppression of inflammation in HFMD patients.

**Method:**

We measured the levels of 50 kinds of chemokines, cytokines, growth factors and soluble receptors in serum samples from control patients without HFMD and the HFMD patients with or without prior treatment of intravenous methylprednisolone.

**Results:**

Our present study found that even relatively mild HFMD patients without central nervous system (CNS) complications had elevated serum levels of inflammatory cytokines, including interleukin (IL)-3, IL-6, IL-12p40, and tumor necrosis factor (TNF)-α, which suggested systemic inflammation. In contrast, these patients also have decreased levels of other serum biomarkers, including IL-1Ra, IL-8, IL-16, soluble ICAM-1, CXCL-1, and CCL27. The dysregulation of cytokine and chemokine expression may be involved in CNS complications and unbalanced circulating leukocytes in HFMD patients. Surprisingly, patients treated with methylprednisolone had no difference in the expression levels of HFMD-associated biomarkers instead had slightly increased levels of IL-17A, which was not associated with the occurrence of HFMD.

**Conclusion:**

Whether steroid treatment has any beneficial effect on the prognosis of HFMD patients requires to be further investigated.

## Author Summary

Systemic inflammation is characteristic of severe hand, foot, and mouth disease (HFMD). Steroids are considered immunomodulators and have been officially recommended to treat the severe HFMD patients with CNS complications in China. So far, it is uncertain whether steroid treatment has an immunomodulatory role in inflammation in HFMD patients and has a real beneficial effect on their prognosis. This study revealed that even relatively mild HFMD patients without CNS complications had elevated inflammation. Unexpectedly, the inflammatory cytokine levels in patients treated with methylprednisolone, one kind of steroid, were not significantly different from those in patients without the treatment. Rather, the treated patients tended to have elevated levels of IL-17A, whose expression levels were actually not significantly associated with the presence of HFMD. IL-17A is known to play a role in the pathogenesis of CNS-related inflammatory diseases. Altogether, our study does not support the presumption that steroids have beneficial effect on the prognosis of HFMD patients by inhibiting systemic inflammation.

## Introduction

Hand, foot and mouth disease (HFMD) is an infectious disease caused by enteroviruses including enterovirus 71 (EV71) and Coxsackievirus A16. HFMD affects mainly young children under 5 years old. The first large EV71 outbreak occurred in Japan in 1973 [1]. Another two large outbreaks subsequently occurred in Hungary and Bulgaria [2], [3]. Even larger outbreaks later occurred in Malaysia in 1997 and subsequently in Taiwan in 1998 [4], [5]. Since 2008, over 6.5 million cases including over 1300 fatalities have been reported in China according to the official report from China Ministry of Health. Forty-eight HFMD cases in Thailand and 411 cases in Vietnam have also been reported in 2008–2009 [6], [7]. In last April, 50 fatalities and at least 60 cases were reported in Cambodia [8]. So far, HFMD has become a great threat to public health in developing countries in Asia.

EV71 is the most neurovirulent virus among the enteroviruses associated with HFMD and causes severe CNS complications and fatal outcome [9], [10]. The CNS manifestations include aseptic meningitis, poliomyelitis-like syndrome, encephalomyelitis, autonomic nervous system (ANS) dysregulation, and brain stem encephalitis (BE). Patients with severe CNS complications are likely to progress to fatal pulmonary edema (PE) and die from cardiopulmonary collapse if timely interventions and advanced life support are not initiated.

Studies of the critically severe HFMD patients with PE suggest that inflammatory cytokine storm may be involved in the pathogenesis of severe CNS complications caused by EV71 infection. For example, infiltration of inflammatory cells has been observed in the brain stem and spinal cord of patients who died of PE [11]–[13]. Consistently, it has been found that IL-6, IL-10, IL-13, interferon (IFN)-γ, and monokine induced by IFN-γ (MIG; CXCL9) express at significantly higher levels in the cerebrospinal fluid of the PE patients than in those with isolated BE [14]–[16]. In addition, systemic levels of IFN-γ-induced protein (IP)-10, IL-6, IL-10, monocyte chemoattractant protein (MCP)-1, and CXCL9 are also significantly higher in patients with PE than in those with uncomplicated BE [15], [17]. The serum levels of IL-1, IL-6, and TNF-α are generally higher in CNS-complicated patients than in patients with uncomplicated HFMD or healthy controls [17]. One unexplained manifestation in PE patients is that they have significantly fewer circulating T cells and natural killer (NK) cells but more neutrophils [16].

Local or systemic inflammation has been implicated at least partly for the increased pulmonary vascular permeability, which may result in development of PE or pulmonary hemorrhage [16], [18], [19]. Thus, intravenous immunoglobulin has been administrated as an immunomodulator to severe HFMD patients at high risk of progressing to PE [18]. In addition, milrinone therapy has been shown to reduce plasma levels of IL-13 and concomitantly sympathetic hyperactivity, suggesting that it has immunomodulatory effect [18]. Since 2008, glucocorticoids, including hydrocortisone, dexamethasone, and methylprednisolone, have been empirically used to treat severe HFMD patients with CNS complications and PE. In a consensus statement of HFMD and EV71 infection in China issued in 2010 by an expert panel, glucocorticoids were also recommended for use in patients developing severe CNS complications and PE (http://www.jkb.com.cn/htmlpage/12/123560.htm?docid=123560&cat=null&sKeyWord=null). Despite the controversy on the rationale and benefits of glucocorticoid therapy in severe enterovirus infection or other viral infections [20]–[22], glucocorticoids indeed have been widely used in the management of severe HFMD with CNS complications in China [23]–[26]. However, little is known so far about the immunomodulatory effects and clinical benefits of glucocorticoid treatment in HFMD patients.

This study systematically analyzed serum levels of inflammatory cytokines, chemokines, growth factors, and soluble immune receptors in patients with either uncomplicated HFMD or severe HFMD complicated with CNS involvement without fatal PE. This study also evaluated the inflammatory responses of HFMD patients treated with methylprednisolone. Our results revealed that even the patients without CNS complications had a distinct expression profile of cytokines, chemokines, growth factors, and soluble immune receptors in their sera as compared with control patients. None of these HFMD-characteristic biomarkers in patients with methylprednisolone treatment differed from those in the patients without the treatment, suggesting that methyprednisolone may not affect the inflammation caused by HFMD.

## Materials and Methods

### Ethics statement

This study was approved by the ethics committee of the Children's Hospital of Fudan University. Written informed consents were obtained for the use of serum samples from all patients (or their parents/guardians) involved in this study.

### Case definition

The criteria of case definition were based on previous reports [16], [27]–[30]. HFMD was characterized by the manifestation of oral ulcers/vesicular plus vesicular rash on the hands, feet, or/and buttocks. Aseptic meningitis was diagnosed on the basis of the presence in CSF of more than 10×10^6^ leukocytes/L with normal glucose, normal or mildly elevated protein, negative results on Gram stain smear, and signs of fever, vomiting, headache, irritability, and meningeal signs in various combinations without altered levels of consciousness or focal signs. Encephalitis was defined as disturbed consciousness plus CSF pleocytosis (>10×10^6^ leukocytes/L) or presence of focal neurologic signs, including abducens palsy, facial palsy, dysphagia, upward gaze, and nystagmus.

### Study subjects and serum samples

We prospectively enrolled 20 HFMD cases with CNS-involvement and 20 HFMD cases without CNS- involvement in late July–September, 2010. The inclusion criteria for the enrollment were as the following: 1) children were hospitalized solely for HFMD within 4 days after disease onset; 2) children were <5 years old; 3) children's parents gave informed consent to participate in this study; 4) children had stool taken for enterovirus test; 5) severe HFMD cases were confirmed based on the clinical presentation and the abnormal findings of cerebral spinal fluids. Gender difference and methylprednisolone treatment were not in our initial consideration for enrollment. Serum samples were taken from the HFMD patients admitted to the infectious disease wards and 20 non-HFMD patients who had minor operations for inguinal hernia or hydrocele at the surgical wards. EV71 infection was diagnosed based on detection of virus in stool specimens. Stool samples from all patients were tested for the presence of enterovirus infection by real-time RT-PCR assay using a commercial kit (Da An Gene Co., Ltd. Lot No.: EV-A71 YZB- 0356-2009).

### Cytokine array

The expression of cytokines, chemokines, growth factors, and soluble immune receptors was examined using Bio-Plex Pro Human Cytokine 27-plex and Bio-Plex Pro Human Cytokine 23-plex kits according to the manufacturer's instructions (BioRad, CA, USA). The detection limits of these parameters were in line with the manufactory instruction.

### Statistical analysis

Proportional data were analyzed using *X* ^2^ or Fisher's exact tests. Continuous data were tested by Student's *t* test to determine the statistical significance of differences. Of note, the undetectable results were replaced with zeroes before the statistical analysis. The P values were further adjusted using the Benjamini-Hochberg method to control for multiple comparison false discovery [31]. The correlations between clinical parameters and biomarkers in the serum samples were evaluated with Spearman's rank correlation test. All analyses were performed using the SPSS software (version 11.5; SPSS). A difference with P<0.05 was considered to be significant.

## Results

### Patient characteristics and clinical symptoms

Among the 40 HFMD patients, 20 were confirmed with meningitis and encephalitis and the remaining 20 had no CNS complications. The age of the 20 control patients were aged between 15 and 56 months (mean age: 37.6 months) with a male-to-female ratio of 1∶1. The age of the 20 patients with uncomplicated HFMD were between 8 and 47 months (mean age: 26.5 months) with a male-to-female ratio of 19∶1. The age of the 20 patients with CNS-complicated HFMD were aged between 11 and 56 months (mean age: 29.0 months) with a male-to-female ratio of 13∶7.

Thirty (72.5%) of the HFMD patients, were EV71-positive in stool specimens by RT-PCR assay (Table 1). Eleven (55%) of 20 uncomplicated HFMD patients and 19 (95%) of 20 severe HFMD patients with CNS involvement were EV71-positive. The severe CNS-complicated patients tended to have fewer white blood cells (WBC) and lymphocytes. All HFMD patients presented with fever. Although the maximal fever temperature was not different between the two groups of HFMD patients, the CNS-complicated patients had significantly longer fevers in average than the uncomplicated (4.2 vs. 2.8 days, P<0.0001). As compared with the uncomplicated patients, the CNS-complicated also had a significantly higher proportion of CNS involvement-related symptoms, such as vomiting, tachycardia, myoclonus, and lethargy (Table 1).

**Table 1:** Clinical characteristics of HFMD patients.

	Mild	CNS-complicated	
Characteristics	(n = 20)	(n = 20)	P value
EV71 infection	55% (11/20)	95% (19/20)	<0.01^a^
Median age, (months) (range)	26.50 (8–47)	29.00 (11–56)	0.4689
Gender (female/male)	1/19	7/13	<0.05^a^
Time of illness at sampling, (day) (range)	1.9 (0–4)	2.0 (0–6)	0.9367
Median WBC, (/mm^3^) (range)	13.52 (4.2–14.8)	10.19 (5.1–21.3)	0.4962
Median neutrophiles, (%) (range)	43.2 (13–71.2)	51.59 (27.3–73.4)	0.0072
Median lymphocytes, (%) (range)	45.98 (23.2–76.9)	36.9 (16–65.8)	0.0102
Median monocytes, (%) (range)	10.10 (4.3–17)	11.10 (5.2–19.4)	0.9308
Median platelets, (/mm^3^) (range)	314.05 (169–561)	323.05 (207–499)	0.8167
Median peak fever temperature, (°) (range)	38.94 (37.8–47)	39.44 (38.7–41)	0.0773
Median fever duration, (days) (range)^b^	2.80 (1–6)	4.20 (3–6)	0.0043
Fever	100% (20/20)	100% (20/20)	>0.05^a^
Oral ulcer	100% (20/20)	100% (20/20)	>0.05^a^
Rash	100% (20/20)	100% (20/20)	>0.05^a^
Vomiting	15% (3/20)	40% (8/20)	>0.05^a^
Tachycardia	5% (1/20)	60% (12/20)	<0.001^a^
Myoclonus	0% (0/20)	45% (9/20)	<0.001^a^
Convulsion	5% (1/20)	0% (0/20)	>0.05^a^
Irritability	0% (0/20)	10% (2/20)	>0.05^a^
Lethargy	0% (0/20)	55% (11/20)	<0.001^a^

WBC, white blood cells.

a , P values were analyzed via *X* ^2^ test; the rest P values were analyzed by Student's *t*-test.

b , fever duration refers to the total duration after hospitalization until the fever had settled.

### The systemic inflammatory profiles of HFMD patients

To gain further knowledge about systemic inflammatory responses in HFMD patients and discover potential biomarkers for disease severity, we examined the expression levels of 50 kinds of cytokines and other immune activation markers in the sera of HFMD patients and control patients with a cytokine array. We found that the expression levels of 24 biomarkers in HFMD patients were not significantly different from those of control patients (Table S1). While 14 biomarkers were significantly elevated (Table 2). Consistent with previous reports [14]–[17], IL-6, IFN-γ, and TNF-α are significantly elevated in the sera of HFMD patients compared to those of controls (Table 2). In addition, IL-2, IL-12p40, IL-15, IL-2Rα, IL-3, eotaxin, IFN-α2, HGF, MCP-3 (CCL7), SCF, and TNF-related apoptosis-inducing ligand (TRAIL) were also significantly increased in the sera of HFMD patients. Of note, IL-2, IL-6, IL-15, TNF-α, and eotaxin were 5- to 10-fold higher in HFMD patients in comparison with the control group. More dramatically, the levels of soluble TRAIL increased approximately 20 times on average, and the levels of IL-3 and IL-12p40 increased approximately 40 times on average.

**Table 2:** Immune biomarkers whose levels in serum samples of HFMD patients were significantly different from those of control patients.

	Control Patients (n = 20)	HFMD Patients (n = 40)	
	Mean ±SD (pg/ml)	Mean ±SD (pg/ml)	P-value*
IL-6	16.39±5.96	164.65±230.63	0.0161
IFN-γ	104.48±47.19	555.09±822.90	0.0407
TNF-α	59.55±18.12	669.56±996.62	0.0213
Eotaxin	55.70±38.72	389.22±415.46	0.0050
IL-12p40	134.93±197.26	6518.19±8257.57	0.0061
IL-15	7.15±6.04	75.18±93.08	0.0068
IL-2	12.58±5.82	163.65±201.37	0.0067
IL-2Rα	522.84±203.72	1746.65±2314.01	0.0474
IL-3	62.12±113.59	2658.45±3544.22	0.0068
IFN-α2	68.90±25.93	280.73±250.45	0.0033
HGF	454.07±150.10	1706.93±2213.63	0.0348
MCP-3	35.44±19.33	285.92±285.41	0.0020
SCF	170.51±74.83	822.27±1007.04	0.0161
TRAIL	70.05±68.02	1476.94±2298.39	0.0213
IL-1Ra	588.02±705.11	185.03±146.76	0.0056
IL-16	3032.39±3095.32	1209.10±1162.22	0.0067
IL-8	54.35±89.05	20.75±15.91	0.0474
M-CSF	41.48±37.54	13.20±18.27	0.0020
MIP-1β	186.61±102.33	123.60±58.64	0.0116
CCL27	1636.82±427.72	1126.67±429.13	0.0016
CXCL1	138.75±67.74	82.79±67.52	0.0116
MIF	13872.58.58±19142.47	4074.63±13558.21	0.0049
PDGF-β	13031.42±4015.56	8131.63±2679.43	0.0008
VEGF	271.87±21.47	168.24±124.72	0.0474
SCGF-β	60051.78±15324.97	38001.95±15318.29	0.0003
VCAM-1	99173.58±23202.09	80279.76±19300.42	0.0067

* Significance was analyzed via Student's *t*-test and P values were further adjusted with the Benjamini-Hochberg procedure.

Unexpectedly, 12 kinds of cytokines and immune activation markers were significantly lower in the HFMD patients than in the control group (Table 2). These markers included IL-1Ra, IL-8, IL-16, PDGF-β, VEGF, MIP-1β, CTACK (CCL27), GROα (CXCL1), M-CSF, SCGF-β, VCAM-1, and MIF. Of them, IL-8, IL-16, IL-1Ra, M-CSF, and MIF were 2–3-fold lower in HFMD patients in comparison with the control group. Altogether, HFMD patients appeared to have systemic inflammation and exhibited a distinct serum inflammatory profile in comparison with control patients.

### The serum levels of VCAM-1 correlated with the maximal fever temperature of HFMD patients

**Figure 1:**
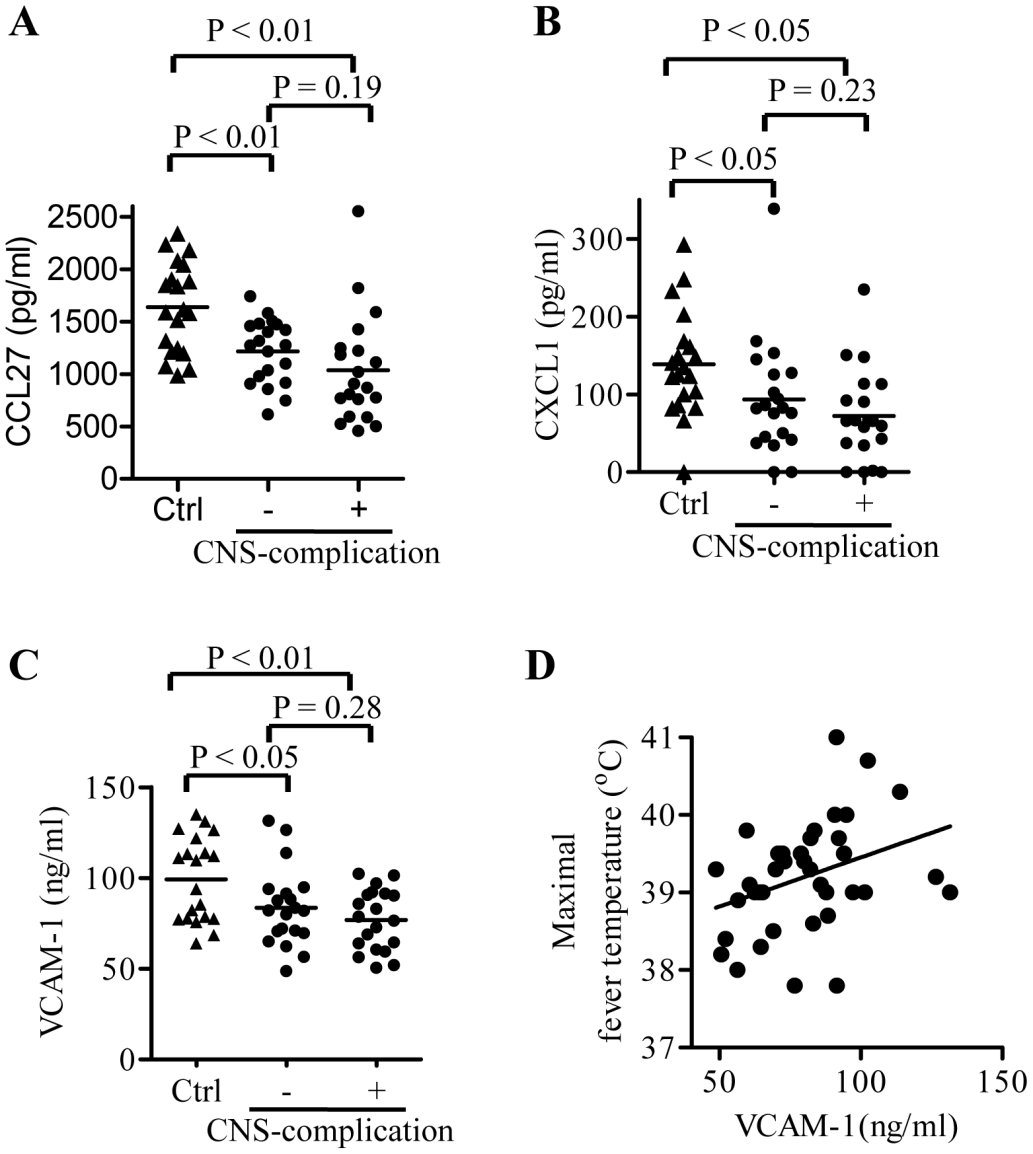
The correlation between host biomarkers and disease prognosis. Levels of CCL27 (A), CXCL1 (B), and soluble VCAM-1 (C) in serum samples obtained from control patients (Ctrl, n = 20) and HFMD patients with or without CNS complications (n = 20, respectively). The line represents the average value. (D) Plots of soluble VCAM-1 concentrations in sera of HFMD patients against their maximal fever temperatures. Numbers above square brackets indicate P values for the corresponding comparisons.

We further analyzed the correlation of the serum biomarker levels with disease severity or prognosis in HFMD patients. We found that upregulated serum biomarkers in CNS-complicated patients did not differ from those in uncomplicated patients. However, we found that the levels of VCAM-1, CXCL-1, and CCL27 in the patients with CNS-complicated HFMD decreased slightly in comparison with those with uncomplicated HFMD (Fig. 1A–C). In particular, VCAM-1 was found to be significantly associated with maximal fever temperature (Fig. 1D).

### Methylprednisolone treatment did not significantly affect the expression of HFMD-characteristic biomarkers but tended to increases serum levels of IL-17 in HFMD patients

**Figure 2:**
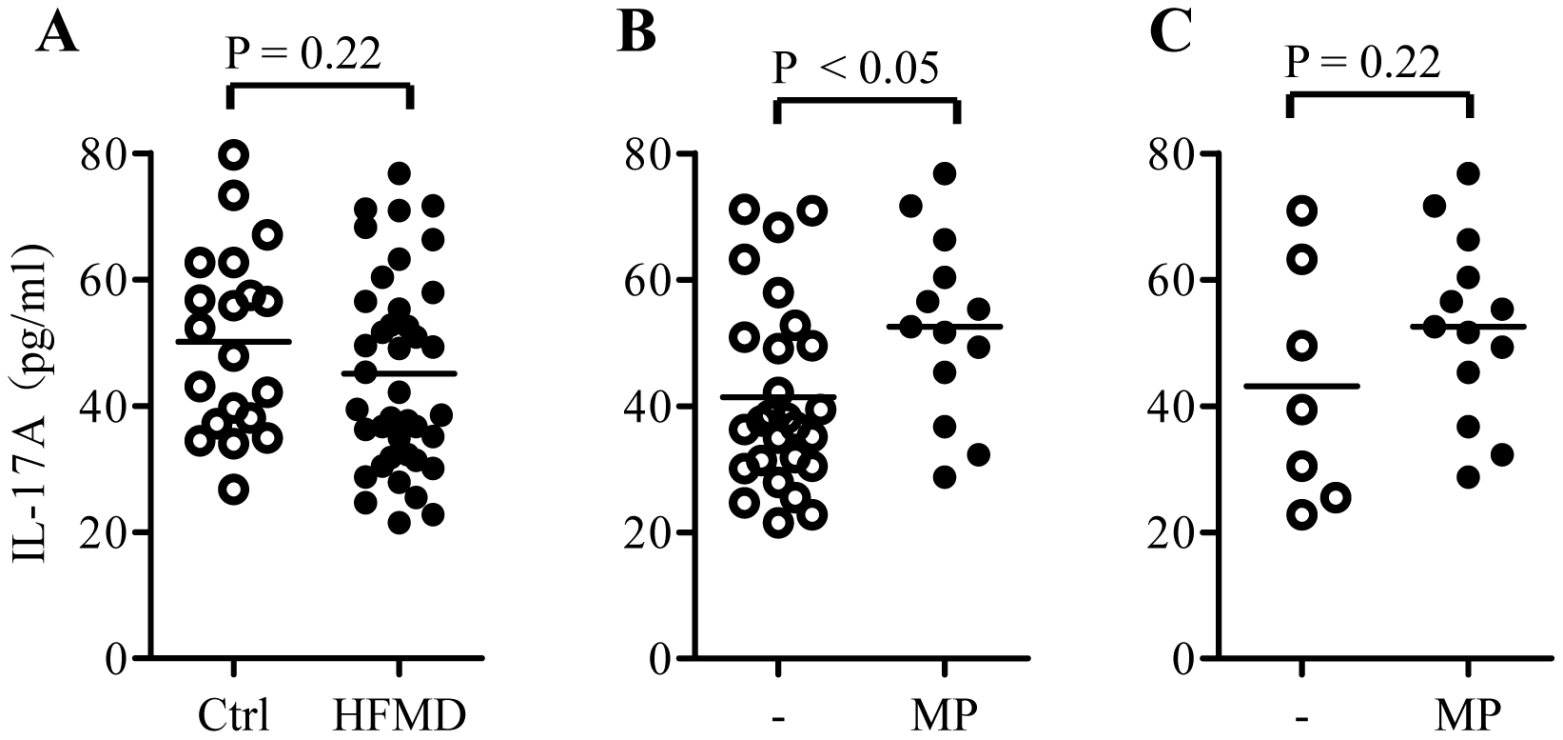
The effect of methylprednisolone (MP) treatment on serum levels of IL-17A. (A) IL-17A levels in serum samples obtained from control (Ctrl, n = 20) and HFMD patients (HFMD, n = 40). (B) IL-17A levels in serum samples obtained from untreated (n = 27) or MP-treated HFMD patients (n = 13). (C) IL-17A levels in serum samples obtained from untreated (n = 7) or MP-treated CNS-complicated HFMD patients (n = 13). Numbers above square brackets indicate P values for the corresponding comparisons.

Among the patients with CNS-complicated HFMD, 13 patients (65%) received at least one dose of methylprednisolone (2–3 mg/kg intravenously) 12 hours prior to blood sampling. We further analyzed the effect of methylprednisolone treatment on the expression of the serum biomarkers. To our surprise, none of HFMD-characteristic inflammatory biomarkers (list in the Table 2) in the treated patients significantly differed from those in the untreated patients (data not shown), implying that methylprednisolone treatment is not effective in inhibiting inflammation caused by HFMD. IL-17A levels in the serum samples of HFMD patients were not different from those of the controls (Fig. 2A). In addition, IL-17A levels in the serum samples of HFMD patients with CNS-complications were not significantly different from the CNS-uncomplicated patients either. Yet, 13 methylprednisolone-treated patients had significantly higher IL-17A levels than the rest HFMD patients when all 40 patients were not stratified by disease severity (Fig. 2B). When patients with CNS-complicated HFMD were further divided into methylprednisolone-treated or untreated groups, the treated group still had slightly higher IL-17A levels than the untreated group (Fig. 2C).

## Discussion

We found that systemic levels of 26 kinds of cytokines, chemokines, soluble receptors, and growth factors differed between patients with HFMD and control patients without HFMD. The increased expression levels of IL-6, IFN-γ, and TNF-α in HFMD patients are consistent with previous studies by others [15], [17], [32]. In addition, this study found that the serum levels of IL-2, IL-15, IL-3, IL-12p40, eotaxin, and soluble TRAIL in HFMD patients increased 5 to 48 times. These observations suggest that HFMD patients had generally elevated inflammation.

Griffiths et al. recently found that IL-1β, IL-1Ra, and granulocyte colony-stimulating factor (G-CSF) were significantly elevated in HFMD patients with cardio-respiratory dysfunction [33]. The IL-1Ra levels were actually decreased and G-CSF levels were not changed significantly in our HFMD patients in comparison to control patients. This discrepancy makes one wonder whether the expression levels of biomarkers are specifically associated with different nature of HFMD-related complications. Therefore, the dramatic increase of soluble TRAIL, IL-3 and IL-12p40 in this study highlights a need for further investigation into their role in the pathogenesis of HFMD and their potential roles as biomarkers for predicting disease progression.

The serum expression levels of 26 serum biomarkers in HFMD patients were significantly different from those in control patients, but their expression levels were not associated with the presence of CNS complications in present study. This result is not exactly consistent with the previous findings that the significantly higher levels of inflammatory cytokines were mainly found in fatal PE patients but not in severe patients without PE [16], [18], [19]. Nevertheless, the change of some biomarkers, like IL-1Ra [33], may indicate the poor prognosis of HFMD patients.

A previous study found that HFMD patients with PE have significantly lower numbers of CD4+ T cells, CD8+ T cells, and NK cells but higher numbers of neutrophils in peripheral blood than HFMD patients without PE [16]. The present study also revealed that even the relatively mild HFMD patients without complications had significantly more neutrophils and fewer lymphocytes than the control group. The increased levels of chemokines MCP-3 and CXCL9 and the decreased levels of MIP-1β, CCL27, and CXCL1 in this study imply that the altered numbers of leukocytes in peripheral blood may result from the differential chemotactic functions of these chemokines. In particular, CXCL9 is an inducible T-cell chemoattractant that is regulated by IFN-γ and mediates the recruitment of effector T cells to sites of inflammation [34]. The decreased number of T cells and NK cells may result from sequestration from the peripheral blood to infected tissue sites because of systemic increased levels of CXCL9, which is fueled by systemic increased IFN-γ. The dysregulation of other chemokines may contribute to the higher number of circulating neutrophils.

To our surprise, none of the 26 HFMD-characteristic biomarkers in the methylprednisolone-treated patients differed from those in untreated patients. Nevertheless, the treatment had a tendency to increase the expression levels of IL-17A (one of the other 24 serum biomarkers) in the CNS-complicated HFMD patients. IL-17A as a Th17 cytokine plays a pathogenic role in CNS-related inflammation, such as multiple sclerosis [35]. Thus, it is unlikely that HFMD patients benefit from methylprednisolone treatment through its induction of IL-17A.

Steroids, such as methylprednisolone, have been also recommended to treat critical severe patients who were infected with H5N1 influenza virus, SARS coronarvirus or pandemic H1N1 influenza virus [20], [36], [37]. The treatment indeed significantly reduced the plasma levels of IL-8, MCP-1 and IP-10 in patients infected with SARS coronarvirus [38]. In addition, short-period steroid treatment appeared to have beneficial clinical effect on the severe patients infected with H1N1 influenza virus or SARS coronavirus [36], [37]. However, it was also reported that steroid treatment had no effect on H5N1-infected patients' survival [20]. Therefore, beneficial or side effects of steroid treatment of HFMD patients require to be further investigated.

At least three limitations exist in the present study. First, the patients were enrolled prospectively for studying biomarkers associated with HFMD and its disease severity. Gender difference was not considered in the initial enrollment of patients. As a result, majority of mild patients were male and had a bias sex ratio in comparison to the severe patients. One of possible reasons is that the male patients were preferentially hospitalized during the enrollment. Nevertheless, there was no significant difference in the cytokine and chemokine expression profiles between male patients and female patients (data not shown). Second, the effect of methylprednisolone treatment was analyzed retrospectively, thus the observations require further justification in a prospective study. Third, limited number of patients with CNS-complications made us impossible to analyze the biomarkers associated with the most critical CNS-complication with cardio-respiratory dysfunction. Further investigation using a large cohort with various CNS complications will likely overcome the limitations.

## Supporting Information

Checklist S1 STROBE Statement—checklist of items that should be included in reports of observational studies.
(DOC)

Table S1 Immune biomarkers whose levels in serum samples of HFMD patients were not significantly different from those of control patients.
(DOC)
