# Supplementary material for: The Cytokine and Chemokine Profiles in Patients with Hand, Foot and Mouth Disease of Different Severities in Shanghai, China, 2010
Source: PLoS Negl Trop Dis. 2013 Dec 19;7(12):e2599. doi: 10.1371/journal.pntd.0002599 (PMC3868519; doi:10.1371/journal.pntd.0002599)
Supplement: Table S1 — Immune biomarkers whose levels in serum samples of HFMD patients were not significantly different from those of control patients. (DOC) [file pntd.0002599.s002.doc]

**Table S1. Immune biomarkers whose levels in serum samples of HFMD patients were not significantly different from those of control patients.**

|  | | Control Patients (n=20) | HFMD Patients (n=40) | |  |  | | |  |  |
| --- | --- | --- | --- | --- | --- | --- | --- | --- | --- | --- |
|  | | Mean ±SD (Median, Range) (pg/ml) | Mean ±SD (Median, Range) (pg/ml) | | |  | | P-value^*^ | | |
| IL-1β | 4.42 ± 7.60(6.02，1.57-23.08) | | | 25.53 ±47.82(37.67, 3.27-232.32)^#^ | | | 0.0640 | | |  |
| IL-4 | 9.69 ± 2.27 (9.93, 5.74-13.72) | | | 10.13 ± 5.23 (8.48, 4.38- 25.44) | | | 0.7640 | | |  |
| IL-5 | 0.37 ± 1.65 (0, 0-7.4) | | | 5.21 ± 11.04 (0, 0- 62.48) | | | 0.0873 | | |  |
| IL-7 | 13.75 ± 2.73 (14.06, 9.55-19.96) | | | 27.68 ± 27.52 (18.88, 9.90-130.88) | | | 0.0530 | | |  |
| IL-9 | 47.51 ± 13.36 (46.54, 26.61-69.92) | | | 48.77 ± 21.9 (43.33, 17.6-105.65) | | | 0.8307 | | |  |
| IL-10 | 8.05 ± 4.07 (6.06, 2.35-14.2) | | | 19.42 ± 23.90 (21.66, 5.13-123.42) | | | 0.6612 | | |  |
| IL-12 | 54.69 ± 28.79 (52.32, 17.45-110.16) | | | 87.02 ± 92.91 (53.39, 7.29- 106.11) | | | 0.1879 | | |  |
| IL-13 | 11.54 ± 4.69 (12.48, 9.3-17.13) | | | 19.72 ± 20.33 (13.57, 7.29-106.11) | | | 0.1174 | | |  |
| IL-17 | 50.14 ± 14.55 (50.1, 34.03-79.74) | | | 45.05 ± 15.4 (40.78, 21.48-71.66) | | | 0.2675 | | |  |
| bFGF | 26.48 ± 13.32 (26.44, 11.50-52.28) | | | 32.02 ± 32.69 (23.71, 12.86-191.61) | | | 0.5234 | | |  |
| G-CSF | 57.78 ± 117.08 (0, 0-457.53) | | | 27.59 ± 42.17 (12.05, 14.51-178.84) | | | 0.2018 | | |  |
| GM-CSF | 25.91 ± 33.6 (9.91, 3.71-123.93) | | | 67.48 ± 139.85 (12.93, 5.21-732.36) | | | 0.2490 | | |  |
| IP-10 | 900.74 ± 364.27 (792.13, 385.75-1267.73) | | | 1371.54 ± 1073.66 (1043.78, 218.93-5331.41) | | | 0.0918 | | |  |
| LIF | 13.10 ± 13.25 (13.17, 4.33-50.73) | | | 29.32 ± 32.58 (30.95, 6.85-140.88) | | | 0.0792 | | |  |
| MCP-1 | 76.09 ± 25.52(77.73, 39.64-142.91) | | | 94.07 ± 82.82 (60.97, 13.47-327.13) | | | 0.3960 | | |  |
| MIG | 1514.22 ± 936.73 (1225.47, 718.34-4515.79) | | | 11188.91 ± 21419.93(16304.42, 533.36-93769.93) | | | 0.0640 | | |  |
| RANTES | 8463.01 ± 8202.98 (6773, 4607.45-31327.85) | | | 5992.71 ± 5035.19(4844.81, 3267.71-33346.12) | | | 0.2205 | | |  |
| IL-1a | 0 ±0 (0,0-0) | | | 0.04 ± 0.17 (0, 0-0.87) | | | 1 | | |  |
| IL-18 | 158.71 ±101.39 (147.17, 60.23-503.71) | | | 213.35 ±178.43 (162.06, 34.73-483.29) | | | 0.2561 | | |  |
| b-NGF | 1.3 ± 2.86 (0, 0-9.97) | | | 37.84 ±83.96 (9.24, 0-416.10) | | | 0.0873 | | |  |
| SDF-1a | 119.72 ±109.28 (129.28, 45.69-376.81) | | | 103.66 ±197.35 (33.44, 6.41-1010.98) | | | 0.7672 | | |  |
| MIP-1a | 10.75 ± 3.53(9.39, 6.70-20.31) | | | 12.34 ± 5.43(11.52, 4.30-21.73) | | | 0.2814 | | |  |
| TNF-b | 8.69 ± 7.96 (9.55, 3.56-20.88) | | | 7.18 ± 8.18 (4.71, 3.39-30.43) | | | 0.5412 | | |  |
| ICAM1 | 286185.99 ±303057.89 (217931.8, 115070.3-1550000) | | | 220454.06 ±77812.54 (198787.1, 87933.4-404094.9) | | | 0.2490 | | |  |

* Significance was analyzed via Student’s *t-*test and p values were further adjusted with the Benjamini-Hochberg-Yekutieli procedure.
